# Vegetarian diet and healthy aging among Chinese older adults: a prospective study

**DOI:** 10.1038/s41514-025-00213-4

**Published:** 2025-04-01

**Authors:** Guliyeerke Jigeer, Kaiyue Wang, Yuebing Lv, Katherine L. Tucker, Xiuhua Shen, Fan Chen, Liang Sun, Xiaoming Shi, Yaqi Li, Xiang Gao

**Affiliations:** 1 Department of Nutrition and Food Hygiene, School of Public Health, Institute of Nutrition, Fudan University, Shanghai, China; 2 China CDC Key Laboratory of Environment and Population Health, Beijing, China; 3 Department of Biomedical and Nutritional Sciences, University of Massachusetts Lowell, Lowell, MA USA; 4 Department of Clinical Nutrition, Xinhua Hospital, Shanghai Jiao Tong University School of Medicine, Shanghai, China

**Keywords:** Epidemiology, Cognitive ageing, Emotion, Risk factors

## Abstract

Vegetarian diets are increasingly popular worldwide, but their impact on healthy aging in older adults remains unclear. This study examined the association between vegetarian diets and healthy aging among 2,888 healthy older Chinese adults from the Chinese Longitudinal Healthy Longevity Survey. Dietary patterns (vegan, ovo-vegetarian, pesco-vegetarian, omnivorous) were derived from a simplified non-quantitative food frequency questionnaire. Over a median follow-up of 6 years, after accounting for sociodemographic and lifestyle factors, vegetarians had lower odds of achieving healthy aging compared to omnivores (adjusted OR = 0.65, 95% CI: 0.47-0.89), with consistent results across sensitivity analyses and individual health components. Additionally, the health effects of vegetarian diets may vary depending on diet quality, with vegetarians of higher diet quality not significantly differing in terms of overall healthy aging and individual outcomes when compared to omnivores. Accordingly, this finding highlights modest inclusion of animal-based foods may improve the overall health status of healthy older adults.

## Introduction

Vegetarian diets, defined as dietary patterns solely/mostly composed of plant-based foods, excluding specific or all animal-derived foods from the diet, have become increasingly popular^1,2^. Several studies have shown that vegetarian diets were associated with lower risks of developing cardiovascular diseases (CVDs), obesity, diabetes, and cancer in young and middle-aged adults^3,4^. Furthermore, vegetarian diets have been recognized for their benefits in mitigating conditions such as metabolic syndrome, obesity, and hyperlipidemia^5^. However, few studies have examined the potential health outcomes of vegetarian diets in older adults – a vulnerable group at risk of malnutrition when following a strict vegetarian diet due to the potential inadequate intakes of protein, vitamin B12, calcium, iron, and essential omega-3 fatty acids^3^. Given age-related physiological changes in digestive and metabolic systems among older adults, vegetarian diets may result in muscle loss and bone fracture, key contributors to physical disability^6–8^. Additionally, evidence regarding the associations between vegetarian diets and other health outcomes, such as mortality, major chronic diseases, cognitive function, and mental health in older adults, is scarce and remains controversial^9–11^. In this context, comprehensively examining the potential impact of vegetarian diets on overall health, especially among older adults, has important clinical and public health implications. Approaches to achieve healthy aging – commonly defined as maintaining disease-free and physically, mentally, and cognitively healthy – are urgently needed, particularly given the low prevalence of healthy aging, which range from 10% to 35% among older adults^12–14^. Identifying modifiable lifestyle factors for healthy aging can help to develop feasible intervention strategies to promote this outcome.

Here, incorporating the definition of healthy aging^12,13,15^, we leveraged longitudinal follow-up data from the Chinese Longitudinal Healthy Longevity Survey (CLHLS) to examine associations between vegetarian diets (including vegan, ovo-vegetarian diet, and pesco-vegetarian diet) and healthy aging, defined as survival to at least 80 years of age with no major chronic diseases (i.e., diabetes, CVDs, stroke, chronic respiratory disease, cancer and hypertension), and no impairment of physical function, cognitive function or mental health. To assess the quality of plant-based foods, we also examined the potential joint effects of vegetarian diets and healthy vs. unhealthy plant-based diet indices (based on the quality of plant-based foods).

## Results

### Study characteristics

The mean age of participants was 72.1 ± 4.35 years and 55.0% were male. Those who committed to the vegetarian diets tended to be female, of the Han ethnic group, with lower income level, and lower education level (Table 1), compared to omnivores.

**Table 1:** Characteristics of participants at baseline by dietary pattern

Characteristic	Omnivore	Vegetarian	*P*-difference^a^
	(*n* = 2512)	(*n* = 376)	
Age			
Mean (SD)	72.1 (4.4)	71.8 (4.3)	0.28
Median [Min, Max]	72.0 [64.0, 79.0]	72.0 [64.0, 79.0]	
Sex, *n* (%)			
Male	1417 (56.4%)	172 (45.7%)	<0.001
Female	1095 (43.6%)	204 (54.3%)	
Ethnic group, *n* (%)			
Han	2316 (92.2%)	366 (97.3%)	<0.001
Other	196 (7.8%)	10 (2.7%)	
Residence, *n* (%)			
Urban	980 (39.0%)	167 (44.4%)	0.07
Rural	1532 (61.0%)	209 (55.6%)	
Marital status, *n* (%)			
Other	889 (35.4%)	133 (35.4%)	1.00
Married	1623 (64.6%)	243 (64.6%)	
Income level, *n* (%)			
<8000 CNY	1993 (79.3%)	302 (80.3%)	0.03
8000–30,000 CNY	360 (14.3%)	53 (14.1%)	
>30,000 CNY	62 (2.5%)	1 (0.3%)	
Missing	97 (3.9%)	20 (5.3%)	
Education years, *n* (%)			
0	1100 (43.8%)	217 (57.7%)	<0.001
1–9 years	1215 (48.4%)	138 (36.7%)	
>9 years	194 (7.7%)	20 (5.3%)	
Missing	3 (0.1%)	1 (0.3%)	
Smoking status, *n* (%)			
Never	1457 (58.0%)	224 (59.6%)	0.78
Former	275 (10.9%)	42 (11.2%)	
Current	780 (31.1%)	110 (29.3%)	
Alcohol use, *n* (%)			
Never	1631 (64.9%)	267 (71.0%)	0.06
Former	179 (7.1%)	25 (6.6%)	
Current	702 (27.9%)	84 (22.3%)	
Exercise status, *n* (%)			
Never	1448 (57.6%)	228 (60.6%)	0.30
Former	61 (2.4%)	12 (3.2%)	
Current	1003 (39.9%)	136 (36.2%)	
BMI, *n* (%)			
<18.5	718 (28.6%)	103 (27.4%)	0.95
18.5–24.0	1331 (53.0%)	201 (53.5%)	
>=24.0	454 (18.1%)	71 (18.9%)	
Missing	9 (0.4%)	1 (0.3%)	

*SD* Standard deviation, *BMI* Body mass index.

^a^Baseline characteristic across the dietary pattern were compared using t-test for continuous variables and chi-square test for categorical variables.

### Vegerarian diets and healthy aging

During a median follow-up period of 6 years, 572 of the 2888 participants met the criteria for healthy aging. Compared to omnivores, vegetarians were less likely to achieve healthy aging, with a multivariable-adjusted odds ratio (OR) of 0.65 (95% confidence interval [CI]: 0.47–0.89). Furthermore, the OR for vegans was 0.43 (95% CI: 0.21–0.89) compared to omnivores (Table 2).

**Table 2:** Odds Ratios (ORs) and 95% confidence intervals (95% CIs) for associations between vegetarian diets and achieving healthy aging

Dietary pattern	Case/n	Base Model^a^	Multivariable-adjusted Model^b^
Omnivore	515/2512	Reference	Reference
Ovo-vegetarian	23/139	0.84 (0.52, 1.35)	0.75 (0.46, 1.23)
Pesco-vegetarian	25/154	0.74 (0.47, 1.18)	0.69 (0.43, 1.10)
Vegan	9/83	0.46 (0.22, 0.95)	0.43 (0.21, 0.89)
Omnivore	515/2512	Reference	Reference
Vegetarian	57/376	0.71 (0.52, 0.97)	0.65 (0.47, 0.89)

^a^Adjusted for age (year) and sex.

^b^Further adjusted for ethnic group (Han vs. others), residence (urban vs. rural), year of education (0, 1–9, > 9 years), household income (<8000 CNY, 8000–30,000 CNY, >30,000 CNY), marital status (married and cohabiting or other), smoking status (never, former, current), alcohol use (never, former, current), exercise status (never, former, current), body-mass index (BMI; underweight, normal, and overweight/obese), and year of enrollment (1998, 2000, 2002, 2005, 2008, 2011 or 2014).

### Dietary pattern changes and healthy aging

Based on dietary pattern change from age 60 years to the study baseline, the multivariable-adjusted OR for those consistently adhering to omnivore diets was 1.78 (95% CI: 1.20–2.64), compared to participants consistently adhering to vegetarian diets. For those who shifted from vegetarian to omnivore diets, the OR decreased to 1.54 (95% CI: 0.98–2.41) (Table 3).

**Table 3:** Odds ratios (ORs) and 95% confidence intervals (95% CI) for associations between change of dietary pattern (from 60 years to baseline) and achieving healthy aging

Changes of dietary pattern	Case/N	Base Model^a^	Multi-adjusted Model^b^
Consistently vegetarian	36/264	Reference	Reference
Consistently omnivore	426/2116	1.59 (1.08, 2.33)	1.78 (1.20, 2.64)
Omnivore to vegetarian	21/112	1.40 (0.76, 2.60)	1.40 (0.74, 2.63)
Vegetarian to Omnivore	89/396	1.54 (0.99, 2.40)	1.54 (0.98, 2.41)

^a^Adjusted for age (year) and sex.

^b^Further adjusted for ethnic group (Han vs. others), residence (urban vs. rural), year of education (0, 1–9, > 9 years), household income (<8000 CNY, 8000–30,000 CNY, >30,000 CNY), marital status (married and cohabiting or other), smoking status (never, former, current), alcohol use (never, former, current), exercise status (never, former, current), body-mass index (BMI; underweight, normal, and overweight/obese), and year of enrollment (1998, 2000, 2002, 2005, 2008, 2011 or 2014).

### Individual components analyses

In the analysis of individual components of healthy aging among participants who survived to age 80 years (*n* = 1582), vegetarians were more likely to have major chronic disease (adjusted OR: 1.60, 95% CI: 1.17–2.18), physical function impairment (adjusted OR: 1.95, 95% CI: 1.25–3.04), and cognitive impairment (adjusted OR 2.05, 95% CI: 1.26–3.33) (Table 4). Additionally, a dose-response relationship was observed across the vegetarian diets (vegan and ovo-vegetarian), and the number of unmet healthy aging criteria (0-4) (*P*-trend <0.05 for all). Likewise, those adhering to vegetarian diets since 60 years of age had higher risks of major chronic diseases and physical and cognitive impairment (Table 4).

**Table 4:** Odds Ratios (ORs) and 95% confidence intervals (95% CI) for associations between dietary patterns and individual components of healthy aging (with 1582 participants alive at age 80 years)^a^

Dietary pattern	Major chronic disease^b^ (case=597)	Mental health impairment^c^ (case=552)	Physical function impairment^d^ (case=154)	Cognitive impairment^e^ (case=120)	Number of Unmet Healthy Aging Criteria
Omnivore (*N* = 1379)	Reference	Reference	Reference	Reference	Reference
Ovo-vegetarian (*N* = 80)	1.34 (0.84, 2.15)	1.00 (0.60, 1.66)	2.28 (1.23, 4.25)	3.10 (1.63, 5.88)	1.31 (1.08, 1.58)
Pesco-vegetarian (*N* = 78)	1.85 (1.15, 2.99)	0.70 (0.40, 1.23)	2.27 (1.18, 4.37)	1.02 (0.41, 2.51)	1.18 (0.98, 1.43)
Vegan (*N* = 45)	1.69 (0.91, 3.12)	1.49 (0.77, 2.88)	0.97 (0.33, 2.82)	2.31 (0.91, 5.88)	1.31 (1.03, 1.68)
Omnivore (*N* = 1,379)	Reference	Reference	Reference	Reference	Reference
Vegetarian (*N* = 203)	1.60 (1.17, 2.18)	0.96 (0.69, 1.36)	1.95 (1.25, 3.04)	2.05 (1.26, 3.33)	1.26 (1.11, 1.42)
Consistently omnivore (*N* = 1141)	Reference	Reference	Reference	Reference	Reference
Consistently vegetarian (*N* = 147)	1.79 (1.25, 2.57)	1.10 (0.74, 1.63)	2.25 (1.37, 3.7)	1.97 (1.12, 3.47)	1.36 (1.18, 1.57)
Omnivore to vegetarian (*N* = 56)	0.98 (0.72, 1.33)	1.35 (0.98, 1.86)	1.11 (0.67, 1.85)	1.46 (0.88, 2.43)	1.09 (0.87, 1.36)
Vegetarian to Omnivore (*N* = 238)	1.16 (0.65, 2.05)	0.81 (0.41, 1.57)	1.30 (0.53, 3.23)	2.21 (0.92, 5.33)	1.09 (0.97, 1.22)

^a^Adjusted for age (year) and sex, ethnic group (Han vs. others), residence (urban vs. rural), year of education (0, 1–9, > 9 years), household income (<8000 CNY, 8000–30,000 CNY, >30,000 CNY), marital status (married and cohabiting or other), smoking status (never, former, current), alcohol use (never, former, current), exercise status (never, former, current), body-mass index (BMI; underweight, normal, and overweight/obese), and year of enrollment (1998, 2000, 2002, 2005, 2008, 2011 or 2014).

^b^Having any major chronic disease: diabetes, heart disease, stroke, chronic respiratory diseases, cancer, or hypertension.

^c^The psychological well-being index score (covering aspects of optimism, sense of personal control, conscientiousness, positive feelings about aging, loneliness, anxiety, and loss of self-worth) was used to test mental health impairment, defined as psychological well-being index score < 24 (lowest quartile).

^d^Activities of Daily Living (ADL) was used to test physical function impairment, defined as needing help in bathing, dressing, toileting, getting out of bed, or feeding.

^e^The validated Chinese version of the Mini-Mental State Exam (MMSE) was used to test cognitive impairment, defined as MMSE score < 18.

### Subgroup analyses

No direct associations were observed between the hPDI or uPDI and the likelihood of achieving healthy aging (Supplementary Table 3). However, vegetarians with high uPDI had an adjusted OR of 0.56 (95% CI: 0.37–0.84) for healthy aging, compared to low-uPDI omnivores. Similarly, compared to high-hPDI omnivores, vegetarians with low hPDI had a multi-adjusted OR of 0.54 (95% CI: 0.30–0.97) for achieving healthy aging (Table 5). There was no significant association between low-uPDI vegetarians and high-hPDI vegetarians and healthy aging, compared to their counterparts (Table 5). For the analysis of those individual healthy aging components, compared to low-uPDI omnivores, high-uPDI Vegetarians were more likely to to have major chronic disease (adjusted OR: 1.74, 95% CI: 1.20–2.52), physical function impairment (adjusted OR: 2.69, 95% CI: 1.59–4.57), and cognitive impairment (adjusted OR 3.00, 95% CI: 1.69–5.34). Likewise, the same pattern were also shown for those low-hPDI vegetarians. Additionally, a dose-response relationship was observed across the high-uPDI vegetarians and low-hPDI vegetarians, and the number of unmet healthy aging criteria (0-4) (*P*-trend <0.05 for all) (Supplementary Table 4).

**Table 5:** Joint analysis of plant-based diet index (PDI) and dietary pattern for achieving healthy aging

uPDI	Case/N	Base model^a^	Multivariable-adjusted^b^	*P*-interaction^c^
Low-uPDI Omnivore	304/1,522	Reference	Reference	0.08
Low-uPDI Vegetarian	21/115	1.03 (0.61, 1.72)	0.97 (0.57, 1.65)	
High-uPDI Omnivore	221/990	1.17 (0.95, 1.45)	1.06 (0.85, 1.32)	
High-uPDI Vegetarian	36/261	0.65 (0.44, 0.97)	0.56 (0.37, 0.84)	
**hPDI**		Base model	Multivariable-adjusted	*P*-interaction
High-hPDI Omnivore	216/1100	Reference	Reference	0.19
Low-hPDI Omnivore	299/1412	1.01 (0.82, 1.24)	1.12 (0.90, 1.39)	
Low-hPDI Vegetarian	16/112	0.60 (0.34, 1.07)	0.54 (0.30, 0.97)	
High-hPDI Vegetarian	41/264	0.76 (0.52, 1.12)	0.77 (0.52, 1.14)	

*uPDI* unhealthy plant-based diet index, *hPDI* healthy plant-based diet index.

^a^Adjusted for age (year) and sex.

^b^Further adjusted for ethnic group (Han vs. others), residence (urban vs. rural), year of education (0, 1–9, > 9 years), household income (<8000 CNY, 8000–30,000 CNY, >30,000 CNY), marital status (married and cohabiting or other), smoking status (never, former, current), alcohol use (never, former, current), exercise status (never, former, current), body-mass index (BMI; underweight, normal, and overweight/obese), and year of enrollment (1998, 2000, 2002, 2005, 2008, 2011 or 2014).

^c^Integration was tested by the likelihood ratio test.

### Sensitivity analyses

The robustness of the observed associations in our main analysis was confirmed in sensitivity analyses (Supplementary Table 5). After excluding participants who had been followed for < 2 years and <5 years, respectively, associations were slightly attenuated but remained significant. Similar associations were observed when additionally adjusting for religion activities, redefining the criteria for healthy aging by modifying the cut-offs for the MMSE score and psychological well-being index, and by using Cox hazard regression models.

## Discussion

In this large, prospective cohort study of healthy Chinese older adults, vegetarian diets, particularly vegan diets, were independently associated with lower likelihood of achieving healthy aging. Individuals who maintained omnivorous diets from age 60 years had higher odds of achieving healthy aging, compared to those who consistently followed vegetarian diets. Additionally, the heath impact of vegetarian diets may vary depending on diet quality, as our results indicated that vegetarians with higher diet quality did not significantly differ in terms of healthy aging outcomes compared to omnivores. To the best of our knowledge, this is the first prospective cohort study to investigate the association between vegetarian diets and overall health status among healthy older adults, indicating that adherence to vegetarian diets may not invariably confer the anticipated health benefits within this demographic. Accordingly, this finding highlights the need for a balanced and flexible dietary strategy that incorporates an appropriate amount of animal products, to optimize the health status of healthy older adults.

Existing studies on the health effects of vegetarian diets remain controversial, with much of the research focusing on middle-aged adults and showing heterogeneous findings regarding mortality, major chronic diseases, physical function, and mental health^9,11,16–19^. For example, the Adventist studies from the US showed an association between vegetarian diet and lower risks of all-cause mortality and cardiovascular diseases^10,18^. In contrast, the EPIC-Oxford (UK) and “45 and Up” (Australia) Study failed to find the same associations^17,20^. However, these studies were conducted in Western countries and focused on specific diseases/outcomes without examining the overall health impact of vegetarian diets on older adults. Of note, prior studies did not account for the diet quality within vegetarian patterns, which may partly explain the inconsistent findings regarding the health effects of vegetarian diets.

We found that vegetarian diets were associated with impaired physical function, consistent with previous research^8,21,22^. It is well documented that vegetarian diets may expose older adults to potential nutritional deficiencies. For instance, previous studies demonstrated that vegetarians were at an increased risk of experiencing hip fracture, compared to omnivores^23^. Despite supplementation with vitamin D and calcium, vegetarians exhibited poorer bone health, as measured by calcaneal quantitative ultrasound, compared to omnivores^22^. Similarly, the risk of sarcopenia may be increased among older adults adhering to vegetarian diets, due to the lower bioavailability and quality of plant-based proteins, which were lower in essential amino acids^8^.

We found that vegetarian diets were associated with higher odds of cognitive impairment at the age of 80 years. To date, research on vegetarian diets and cognitive impairment has yielded inconsistent conclusions^9^. Vegetarian diets, being low in saturated fats and cholesterol, have been associated with healthier blood lipid profiles, and they are rich in vitamins, antioxidants, and dietary fiber, potentially offering some protection against cognitive decline^9,24^. However, these diets may lack specific micronutrients, potentially leading to deficiencies in vitamin B12, vitamin D, and docosahexaenoic Acid (DHA) – which are essential for cognitive health^25–27^. Future studies on vegetarian diets and cognitive impairment should expolore supplementation with specific micronutrients to optimize potential benefits.

In our analysis, no significant associations were observed between vegetarian diets and mental health, aligning with the varied findings of existing observational studies on this topic. While several large cross-sectional studies reported higher occurrence of depressive symptoms among vegetarians, compared to non-vegetarians^28,29^, one study found a beneficial association between vegan diets and improved mood disturbances^28^. This inconsistency underscores the need for further investigation.

Our analysis revealed that older adults who consumed vegetarian diets and had poor diet quality, as suggested by lower hPDI scores or higher uPDI scores, had lower odds of achieving healthy aging, and were more likely to experience major chronic diseases, physical function impairments, and cognitive decline, compared to their counterparts with better diet quality. Importantly, the high hPDI and low uPDI vegetarian groups did not exhibit a significantly higher risk of unhealthy aging compared to their counterparts, indicating that a healthier diet overall, regardless of being vegetarian or omnivore, may play a more significant role in promoting healthy aging. These findings highlight the importance of prioritizing overall diet quality even within plant-based dietary patterns. Our results are broadly consistent with previous research that identified associations between lower hPDI / higher uPDI and increased risks of mortality^30^ and several major chronic diseases^31–34^.

The adverse health effect for healthy aging in older adults may be explained by several mechanisms. One explanation could be the higher risk of nutritional deficiencies associated with vegetarian diets in this population, which were essential for maintaining muscle mass, bone health, and cognitive function^3,6^. Age-related physiological changes, such as reduced nutrient absorption, may exacerbate these risks. Notably, protein intake plays a critical role. In our study, older adults following a vegan diet were most strongly associated with adverse effects on healthy aging, compared to those adhering to ovo-vegetarian or pesco-vegetarian diets. Research showed that nearly half of older adults failed to meet the recommended dietary allowance for protein, with primary sources of dietary protein being meat, eggs, dairy, and cereals^35,36^. Vegan diets may increase the risk of protein deficiency due to the lower protein density and suboptimal essential amino acid profiles of plant-based foods. Furthermore, plant proteins are generally of lower quality compared to animal proteins, potentially exacerbating age-related declines in muscle and bone health. These combined factors could significantly impact healthy aging outcomes in older adults^8^. Furthermore, socioeconomic status (SES) played a role in shaping diet quality. For instance, individuals with lower SES may adapt vegetarian diets due to relatively lower price of plant-based diets in China, potentially leading to less diverse and nutrient-rich food intake - factors that contribute to diet quality disparities within plant-based diets^37^. Finally, cultural and ethnic factors may influence dietary patterns, as vegetarian diets in China often differ from those in Western countries, with greater reliance on staple foods and fewer fortified products^38^. These findings underscored the importance of tailoring dietary recommendations to account for individual and contextual factors to promote healthy aging effectively.

Our study has several limitations. First, dietary intake data were collected using a non-quantitative dietary questionnaire, subject to measurement error in diet assessment, making it impossible to adjust for total energy intake. This limitation also means that vegetarians were defined based solely on the frequency of animal product consumption. However, the frequency of intake may be more important than portion size to distinguish between high and low consumption of fruits and vegetables, and several studies have demonstrated the reliability and validity of using non-quantitative FFQ to assess dietary patterns^39,40^. Additionally, the lack of data on dairy consumption precluded us from identifying the ovo-lacto-vegetarian group. Future studies with more detailed and dietary data are needed to better address these distinctions. Secondly, self-report of chronic diseases may lead to an underestimation of disease prevalence and potential misclassification. However, this underestimation is likely global in nature, potentially impacting the study population uniformly^41^. Thirdly, the study’s focus on the Chinese elderly population limits the generalizability of the findings to other ethnic and racial groups, necessitating further research to understand dietary patterns and health outcomes across diverse cultural contexts. Finally, the relatively small sample size of our study may limit the statistical power and stability of our analysis, posing a risk of overfitting, which may reduce the generalizability of the results. Future studies with larger sample sizes are warranted to validate our findings and ensure the robustness of the associations observed in this study.

In conclusion, we observed associations between vegetarian diets and lower likelihood of achieving healthy aging in older adults. Consistently adhering to omnivorous diets was associated with higher likelihood of achieving healthy aging outcomes, compared to those who stably followed vegetarian diets. This implies that judicious control of plant-based food intake and modest introduction of animal-based foods may improve the overall health status of healthy older adults. These results could inform personalized dietary recommendations for older adults, emphasizing the importance of balanced diets that incorporate both plant- and animal-based foods. Older adults adhering to vegetarian diets should be carefully monitored for potential nutrition deficiencies and provided with personalized nutritional advice if indicated. Promoting healthy dietary patterns that support healthy aging may help reduce the burden of age-related diseases and enhance quality of life in aging populations. Furthermore, carefully designed studies are urgently needed to elucidate the potential health benefits and risks of vegetarian diets in this demographic, ensuring that dietary guidelines are evidence-based and tailored to their unique nutritional needs.

## Methods

### Study design and participants

The CLHLS is a nationally representative ongoing longitudinal study initiated in 1998, designed to investigate the determinants of healthy aging. Participants in the CLHLS were aged 60 years and older, and were recruited from 22 of the 31 provinces in mainland China, covering approximately 85% of the population. The CLHLS has systematically collected data across eight survey waves, conducted in 1998, 2000, 2002, 2005, 2008, 2011, 2014, and 2018. Each survey wave involved both follow-up assessments of existing participants and the enrollment of new ones. A detailed description of the study design can be found elsewhere^42^. Ethics approval was obtained from the Research Ethics Committees of Peking University and Duke University (IRB00001052-13074) and signed written consent forms were obtained from all participants or their legal representatives.

At baseline and during follow-up, participants’ health status was evaluated for self-reported major chronic diseases, and for physical, cognitive and mental health performance (details are described in Assessment of Healthy Aging). Participants were considered healthy if no issues were identified in the aforementioned aspects. For the purpose of our study, a total of 6974 (out of 34,436 total) participants, aged between 60 and 79 years at enrollment, were initially included as potential healthy aging candidates (i.e., those who could reach 80 years of age by 2018). We then excluded participants who were lost to follow-up, had missing data on dietary intake at baseline or on healthy aging components during follow-up (*n* = 1205), or did not meet the healthy aging criteria at baseline (*n* = 2881), leaving 2,888 participants for the current analyses (Supplementary Figure 1).

### Assessment of healthy aging

Drawing upon the concept of healthy aging^43,44^, we defined healthy aging as survival to at least 80 years, coupled with no self-reported major chronic disease and no impairment in cognitive function, physical function, or mental health (Supplementary Table 1). Detailed criteria include: (1) no self-reported major chronic disease, including diabetes, CVD, stroke, chronic respiratory disease, cancer, and hypertension, assessed by self-report questionnaire; (2) no physical function impairment, as assessed with the Activities of Daily Living (ADL) index, and defined as not requiring any form of assistance (ranging from partial to complete help) in performing daily tasks such as bathing, dressing, toileting, getting out of bed, or feeding^45^; (3) no cognitive impairment, as assessed with the validated Chinese version of the Mini-Mental State Exam (MMSE), with a score of 18 or higher indicating the absence of cognitive impairment^46^; and (4) no mental health impairment: psychological well-being was measured by seven items covering positive aspects (optimism, sense of personal control, conscientiousness, and positive feelings about aging) and negative aspects (loneliness, anxiety, and loss of self-worth) using a five-point Likert scale. A psychological well-being index was constructed from the sum of these seven items (range 0–35, with higher scores indicating better mental health), and good mental health function was defined as a score ≥ 24 (the lowest quartile)^47^.

### Assessment of vegetarian diet

Information on dietary intake at baseline and recalled habitual intake at 60 years of age was collected using a simplified food frequency questionnaire (FFQ), comprising 12 food group items, through in-person interviews by trained research staff, as detailed previously^48,49^. Although the use of frequency may lack detailed quality, previous studies have shown this non-quantitative form of questionnaire to be reliable and valid. In addition, previous studies also showed that frequency of intake is more important than portion size to distinguish between high and low consumption of fruits and vegetables^39,40,50^. Based on the consumption frequency of the following items: eggs, fish or seafood, and meat (including beef, poultry, pork, lamb, and processed meats), participants were categorized into four types of dietary patterns: the vegan diet (no consumption of eggs, fish, seafood, or meat), the ovo-vegetarian diet (consumption of eggs but no fish, seafood or meat), the pesco-vegetarian diet (consumption of fish or seafood, with or without eggs, but no meat), and the omnivore diet (consumption of meat)^51^. These dietary patterns were further dichotomized into vegetarian and omnivore diets by combining the first three categories, due to their common exclusion of meat and poultry as primary protein sources, in alignment with the broader definition of vegetarianism. As a secondary exposure, dietary pattern change from age 60 y to baseline was examined, and participants were classified into four groups: consistently vegetarian (vegetarian at both age 60 and baseline), vegetarian to omnivore, omnivore to vegetarian, and consistently omnivore (omnivore at both age 60 and baseline).

We further calculated the healthy plant-based diet index (hPDI), and unhealthy plant-based diet index (uPDI), using a method adapted from Satija et al.^52^, with modifications to food group intake frequencies to align with the qualitative nature of the FFQ, to evaluate dietary adherence to healthy/unhealthy plant-based dietary patterns. Twelve food groups were scored from 1 to 5; the animal-based food groups received negative scores, while the healthy plant-based food received positive scores for hPDI and negative scores for uPDI. Detailed scoring information is in Supplementary Table 2.

### Assessment of covariates

Sociodemographic factors, including age (year), sex (male, female), ethnic group (Han, other), residence (urban vs. rural dwellers), years of education (0, 1-9, >9 years), household income (< 8000 CNY, 8000–30,000 CNY, > 30,000 CNY), and marital status (married and cohabiting, or other); and lifestyle factors, including smoking status (never, former, current), alcohol use (never, former, current), exercise status (never, former, current) were collected via questionnaires. Weight and height were measured by trained medical staff during the physical examination. Body mass index (BMI) was calculated as weight (kg) / height squared (m^2^), and categorized as underweight (BMI < 18.5 kg/m^2^), recommended (18.5 kg/m^2^ ≤ BMI < 24.0 kg/m^2^), or overweight/obese (BMI ≥ 24.0 kg/m^2^)^53^. Given that height measurements were unavailable in the first three waves (1998, 2000, 2002) of surveys, knee height was adopted to estimate individual height, using validated equations for older Chinese men (height = 67.78 + 2.01 × knee height) and women (height = 74.08 + 1.81 × knee height)^54^.

### Statistical analysis

All statistical analyses were performed using R version 4.2. The STROBE-NUT reporting guidelines were used to report the checklist for this study (STROBE-NUT checklist)^55^. Baseline characteristics across dietary patterns (vegetarian vs. omnivore) were compared using t-tests for continuous variables and chi-square tests for categorical variables. Covariates with missing data were assigned to an independent category. Similar to a previous study^13^, logistic regression models were used to estimate the likelihood of achieving healthy aging across diet patterns (vegan, ovo-vegetarian, pesco-vegetarian, and omnivore, along with the combined vegetarian vs. omnivore) and status change diets from 60 years (consistently vegetarian, vegetarian to omnivore, omnivore to vegetarian, and consistently omnivore), ORs and 95% CIs were computed. In model 1, we adjusted for age and sex. In model 2, we further adjusted for ethnic group, residence, marital status, household income level, education level, smoking status, alcohol use, exercise status, BMI, and the year of enrollment. In the secondary analysis, we focused on participants who successfully lived to 80 years, and examined how dietary patterns and changes in these patterns were associated with individual health domains: major chronic diseases, cognitive impairment, physical function impairment, and mental health impairment) (*n* = 1585).

Additionally, we assessed the associations of healthful, and unhealthful plant-based dietary patterns with healthy aging, adjusting for the aforementioned covariates. The hPDI and uPDI were used as categorical variables (divided into quintiles) in the analysis, following the approach used in previous studies^56^. To examine interactions between the healthiness of plant-based dietary patterns and vegetarian dietary patterns, we conducted a joint analysis, based on median-divided uPDI score (high uPDI vs. low uPDI) and dietary pattern (vegetarian vs. omnivore), categorizing participants into four distinct groups: high uPDI omnivores, high uPDI vegetarians, low uPDI omnivores, and low uPDI vegetarians. A similar analysis was performed based on hPDI scores. Similar analysis was conducted in those participants who successfully lived to 80 years (*n* = 1585) to examine associations of these patterns with individual health domains.

Four types of sensitivity analyses were carried out to test the robustness of the results: (1) Two- and five-year lag analyses were performed by excluding participants who had been followed for less than 2 or 5 years from baseline, respectively, to reduce the possibility of reverse causation; (2) Additionally adjusted for whether participants engaged in religious activities (only availble in the first three waves); (3) Modifying the definitions of cognitive impairment and mental health impairment by adjusting the cut-off score for the MMSE to 24, and for the psychological well-being index to 27 (median), respectively; and (4) Using Cox Proportional Hazards regression to calculate hazard ratios (HR) and 95% CI for healthy aging across different dietary groups.

## Supplementary information

Supplementary information

## Data Availability

This article is based on a publicly available dataset derived from the Chinese Longitudinal Healthy Longevity Survey (CLHLS). The dataset can be obtained after sending a data user agreement to the data team on the webset of Peking University Open Research Data: https://opendata.pku.edu.cn/dataset.xhtml?persistentId=doi:10.18170/DVN/WBO7LK&version=2.0. Other data that support this work are available from the corresponding author upon reasonable request.

## Abbreviations

CLHLS: Chinese Longitudinal Healthy Longevity Survey;
FFQ: Food Frequency Questionnaire;
MMSE: Mini-Mental State Exam
PDI: Plant-Based Diet Index.
