# Supplementary material for: Vegetarian diet and healthy aging among Chinese older adults: a prospective study
Source: NPJ Aging. 2025 Apr 1;11(1):25. doi: 10.1038/s41514-025-00213-4 (PMC11961757; doi:10.1038/s41514-025-00213-4)
Supplement: Supplementary file 1 — Supplementary information [file 41514_2025_213_MOESM1_ESM.pdf]

Supplementary Materials

Vegetarian diet and healthy aging among Chinese older adults: a prospective study

Table of Content

SUPPLEMENTARY FIGURE 1. PARTICIPANT FLOWCHART 2

SUPPLEMENTARY TABLE 1. DEFINITION AND DIMENSIONS OF HEALTHY AGING. 3

SUPPLEMENTARY TABLE 2. SCORING OF PLANT-BASED DIET INDICES. 5

SUPPLEMENTARY TABLE 3. ODDS RATIOS (95% CIS) OF HEALTHY AGING BY H-PDI AND U-PDI. 8

SUPPLEMENTARY TABLE 4. ODDS RATIOS (ORS) AND 95 % CONFIDENCE INTERVALS (95% CI) OF INDIVIDUAL COMPONENTS OF HEALTHY AGING ACROSS DIFFERENT DIETARY AND PDI GROUPS (WITH 1,582 PARTICIPANTS ALIVE AT AGE 80 Y) [1] 10

SUPPLEMENTARY TABLE 5. SENSITIVITY ANALYSIS FOR ASSOCIATIONS BETWEEN VEGETARIAN DIETS AND ACHIEVING HEALTHY AGING [A] 12

REPORTING CHECKLIST FOR OBSERVATIONAL STUDIES IN NUTRITIONAL EPIDEMIOLOGY. 14

Supplementary Figure 1. Participant flowchart

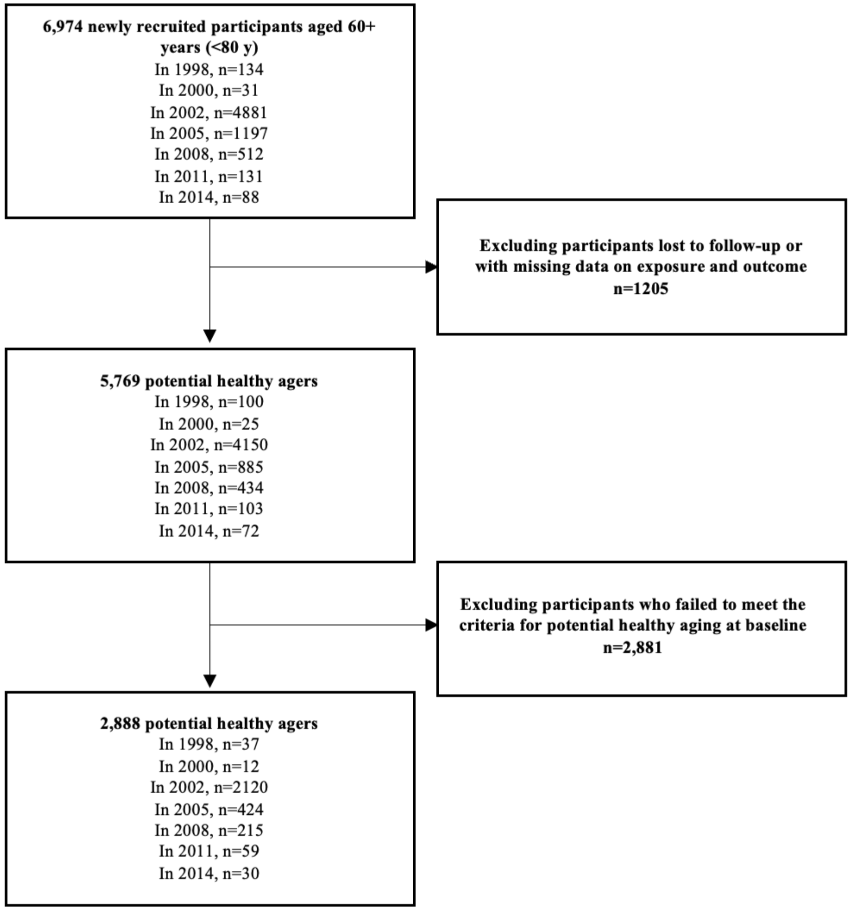

Supplementary Table 1. Definition and Dimensions of Healthy Aging.

Dimension	Healthy aging
Assessment of chronic diseases	Self-report of 6 major chronic diseases (diabetes, heart diseases, stroke, chronic respiratory diseases, cancer, and hypertension)
Assessment of cognitive function	A 30-point validated Chinese version of the Mini-Mental State Exam (MMSE) including five aspects of cognitive functioning (orientation, registration, attention or calculation, recall, and language); no cognitive impairment was defined as MMSE score≥ 18.
Assessment of physical function	The index of Activities of Daily Living (ADL) was available in each wave to test physical function; no physical function impairment was defined as did not need help in bathing, dressing, toileting, getting out of bed, or feeding.
Assessment of mental health	Psychological well-being was measured by seven items covering positive (optimism, sense of personal control, conscientiousness, and positive feelings about aging) and negative (loneliness, anxiety, and loss of self-worth) affects with a five-point Likert scale. A psychological well-being index was constructed from the sum of these seven items (range 0-35, with higher score indicating better mental health); good mental health was defined as score ≥ 24 (the lowest quartile).

Healthy aging	Healthy aging was defined as surviving to the end of follow-up (age ≥80 y), free of major chronic diseases, with no impairment of physical function, no mental health limitations, and no impairment of cognition.

Supplementary Table 2. Scoring of plant-based diet indices.

Type	Food	The first four waves (1998, 2000, 2002, 2005)			The last three waves (2008, 2011, 2014)		
		Frequency	h-PDI	u-PDI	Frequency	h-PDI	u-PDI
Healthy plant-based food	Whole grain	Yes	5	1	Yes	5	1
		No	1	5	No	1	5
	Fresh fruits	Almost everyday	5	1	Almost everyday	5	1
		Except winter	4	2	Except winter	4	2
		Occasionally	2	4	Occasionally	2	4
		Rarely or never	1	5	Rarely or never	1	5
	Vegetables	Almost everyday	5	1	Almost everyday	5	1
		Except winter	4	2	Except winter	4	2
		Occasionally	2	4	Occasionally	2	4
		Rarely or never	1	5	Rarely or never	1	5
	Bean	Almost everyday	5	1	Almost everyday	5	1
					1+ times/week	4	2
		Occasionally	2	4	1+ times/month	3	3
		Rarely or never	1	5	Occasionally	2	4
					Rarely or never	1	5
	Garlic	Almost everyday	5	1	Almost everyday	5	1
					1+ times/week	4	2
		Occasionally	2	4	1+ times/month	3	3

		Rarely or never	1	5	Occasionally	2	4
					Rarely or never	1	5
	Tea	Almost everyday	5	1	Almost everyday	5	1
					1+ times/week	4	2
		Occasionally	2	4	1+ times/month	3	3
		Rarely or never	1	5	Occasionally	2	4
					Rarely or never	1	5
Less healthy plant-based food	Refined grain	Yes	1	5	Yes	1	5
		No	5	1	No	5	1
	Salt-preserved vegetables	Almost everyday	1	5	Almost everyday	1	5
					1+ times/week	2	4
		Occasionally	4	2	1+ times/month	3	3
		Rarely or never	5	1	Occasionally	4	2
					Rarely or never	5	1
	Sugar	Almost everyday	1	5	Almost everyday	1	5
					1+ times/week	2	4
		Occasionally	4	2	1+ times/month	3	3
		Rarely or never	5	1	Occasionally	4	2
					Rarely or never	5	1
Animal-based food	Meat	Almost everyday	1	1	Almost everyday	1	1
					1+ times/week	2	2
		Occasionally	4	4	1+ times/month	3	3
		Rarely or never	5	5	Occasionally	4	4
					Rarely or never	5	5

	Fish	Almost everyday	1	1	Almost everyday	1	1
					1+ times/week	2	2
		Occasionally	4	4	1+ times/month	3	3
		Rarely or never	5	5	Occasionally	4	4
					Rarely or never	5	5
	Egg	Almost everyday	1	1	Almost everyday	1	1
					1+ times/week	2	2
		Occasionally	4	4	1+ times/month	3	3
		Rarely or never	5	5	Occasionally	4	4
					Rarely or never	5	5

Abbreviation: h-PDI = healthy plant-based diet index; u-PDI = unhealthy plant-based diet index.

Supplementary Table 3. Odds Ratios (95% CIs) of Healthy Aging by h-PDI and u-PDI.

**plant-based index**	Base Model [a]	Multivariable-adjusted [b]
h-PDI		
Q1	Reference	Reference
Q2	0.83 (0.62, 1.10)	0.81 (0.61, 1.09)
Q3	1.00 (0.73, 1.36)	0.91 (0.66, 1.25)
Q4	0.91 (0.68, 1.21)	0.82 (0.62, 1.11)
Q5	0.96 (0.71, 1.29)	0.86 (0.63, 1.17)
P-trend	0.91	0.33
u-PDI		
Q1	Reference	Reference
Q2	0.94 (0.7, 1.27)	0.93 (0.68, 1.26)
Q3	0.97 (0.72, 1.31)	0.94 (0.68, 1.28)
Q4	0.95 (0.7, 1.29)	0.85 (0.61, 1.17)
Q5	1.12 (0.82, 1.52)	0.96 (0.69, 1.33)
P-trend	0.54	0.64

Abbreviation: h-PDI = healthy plant-based diet index; u-PDI = unhealthy plant-based diet index.

[a] Adjusted for age (y) and sex

[b] Further adjusted for ethnic group (Han vs. others), residence (urban vs. rural), y of education (0, 1-9, > 9 years), household income (<8,000 CNY, 8000-30000 CNY, >30000 CNY), marital status (married and cohabiting or other), smoking status (never, former, current), alcohol use (never, former, current), exercise status (never, former, current), body-mass index (BMI; underweight, normal, and overweight/obese), and y of enrollment (1998, 2000, 2002, 2005, 2008, 2011 or 2014).

Supplementary Table 4. Odds Ratios (ORs) and 95 % confidence intervals (95% CI) of individual components of healthy aging across different dietary and PDI groups (with 1,582 participants alive at age 80 y) [1]

Dietary pattern	Major chronic disease [2] (case=597)	Mental health impairment [3] (case=552)	Physical function impairment [4] (case=154)	Cognitive impairment [5] (case=120)	Number of Unmet Healthy Aging Criteria
uPDI					
Low-uPDI Omnivore (N=816)	Reference	Reference	Reference	Reference	Reference
Low-uPDI Vegetarian (N=56)	1.20 (0.67, 2.14)	0.89 (0.47, 1.69)	1.42 (0.56, 3.57)	1.39 (0.44, 4.37)	1.07 (0.85, 1.34)
High-uPDI Omnivore (N=563)	0.97 (0.76, 1.23)	1.03 (0.80, 1.32)	1.47 (0.99, 2.18)	1.58 (1.01, 2.46)	1.05 (0.96, 1.15)
High-uPDI Vegetarian (N=147)	1.74 (1.20, 2.52)	1.01 (0.67, 1.51)	2.69 (1.59, 4.57)	3.00 (1.69, 5.34)	1.38 (1.19, 1.60)
hPDI					
High-hPDI Omnivore (N=593)	Reference	Reference	Reference	Reference	Reference
Low-hPDI Omnivore (N=786)	0.93 (0.73, 1.17)	0.93 (0.73, 1.19)	0.78 (0.50, 1.21)	0.78 (0.50, 1.21)	0.96 (0.88, 1.05)
Low-hPDI Vegetarian (N=68)	2.10 (1.24, 3.55)	0.89 (0.50, 1.59)	2.40 (1.16, 4.96)	2.40 (1.16, 4.96)	1.38 (1.12, 1.70)
High-hPDI Vegetarian (N=135)	1.30 (0.88, 1.93)	0.94 (0.61, 1.45)	1.46 (0.74, 2.85)	1.46 (0.74, 2.85)	1.16 (0.99, 1.36)

Abbreviation: h-PDI = healthy plant-based diet index; u-PDI = unhealthy plant-based diet index.

[1] Adjusted for age (y) and sex, ethnic group (Han vs. others), residence (urban vs. rural), y of education (0, 1-9, > 9 years), household income (<8,000 CNY, 8000-30000 CNY, >30000 CNY), marital status (married and cohabiting or other), smoking status (never, former, current), alcohol use (never, former, current), exercise status (never, former, current), body-mass index (BMI; underweight, normal, and overweight/obese), and y of enrollment (1998, 2000, 2002, 2005, 2008, 2011 or 2014).

[2] Having any major chronic disease: diabetes, heart disease, stroke, chronic respiratory diseases, cancer, or hypertension.

[3] The psychological well-being index score (covering aspects of optimism, sense of personal control, conscientiousness, positive feelings about aging, loneliness, anxiety, and loss of self-worth) was used to test mental health impairment, defined as psychological well-being index score < 24 (lowest quartile).

[4] Activities of Daily Living (ADL) was used to test physical function impairment, defined as needing help in bathing, dressing, toileting, getting out of bed, or feeding.

[5] The validated Chinese version of the Mini-Mental State Exam (MMSE) was used to test cognitive impairment, defined as MMSE score < 18.

Supplementary Table 5. Sensitivity analysis for associations between vegetarian diets and achieving healthy aging [a].

		Omnivore	Ovo-vegetarian	Pesco-vegetarian	Vegan	Vegetarian vs. Omnivore
2-year lag analysis [b]	Case/Total	501/2333	23/134	21/139	8/80	52/353
	OR (95% CI)	Reference	0.75 (0.45, 1.23)	0.65 (0.39, 1.09)	**0.38 (0.17, 0.82)**	**0.62 (0.44, 0.87)**
5-year lag analysis [c]	Case/Total	318/1416	17/93	11/84	3/49	31/226
	OR (95% CI)	Reference	0.72 (0.40, 1.30)	0.50 (0.25, 1.01)	**0.18 (0.05, 0.62)**	**0.50 (0.33, 0.78)**
Adjusting for religion [d]	Case/Total	512/2888	23/139	25/154	9/83	57/376
	OR (95% CI)	Reference	0.76 (0.47 ,1.23)	0.70 (0.44 ,1.11)	0.44 (0.21 ,0.90)	0.66 (0.48 ,0.90)
Healthy aging (Modified cognitive function criteria) [e]	Case/Total	473/2512	21/139	21/154	8/83	50/376
	OR (95% CI)	Reference	0.75 (0.45, 1.25)	0.61 (0.37, 1.00)	**0.42 (0.19, 0.90)**	**0.61 (0.44, 0.86)**
Healthy aging (Modified mental function criteria) f	Case/Total	356/2512	13/139	20/154	6/83	39/376
	OR (95% CI)	Reference	0.64 (0.35, 1.17)	0.84 (0.51, 1.39)	0.48 (0.20, 1.14)	**0.69 (0.48, 0.99)**
Cox regression model	Case/Total	515/2512	23/139	25/154	9/83	57/376
	HR (95% CI)	Reference	0.76 (0.50, 1.16)	0.66 (0.42, 1.02)	**0.40 (0.20, 0.79)**	**0.63 (0.47, 0.84)**

[a] Adjusted for age (y), sex, ethnic group (Han vs. others), residence (urban vs. rural), y of education (0, 1-9, > 9 years), household income (<8,000 CNY, 8000-30000 CNY, >30000 CNY), marital status (married and cohabiting or other), smoking status (never, former, current), alcohol use (never, former, current), exercise status (never, former, current), body-mass index (BMI; underweight, normal, and overweight/obese), and y of enrollment (1998, 2000, 2002, 2005, 2008, 2011 or 2014).

[b] Participants who had been followed for less than 2-y from baseline were excluded.
[c] Participants who had been followed for less than 5-y from baseline were excluded.
[d] Further adjusted for participating religious activities (yes vs. no).
[e] Modifying the definition of cognitive impairment by adjusting the cut-off scores of the Mini-Mental State Exam (MMSE) score to 24.
[f] Modifying the definition of mental health impairment by adjusting the cut-off psychological well-being index score to 27 (the median value)

Reporting checklist for observational studies in nutritional epidemiology.

		Reporting Item	Page Number
**Title and abstract**			
Title	#1a	Indicate the study's design with a commonly used term in the title or the abstract	1
None	#nut-1	State the dietary/nutritional assessment method(s) used in the title or in the abstract.	2
Abstract	#1b	Provide in the abstract an informative and balanced summary of what was done and what was found	2-3
**Introduction**			
Background / rationale	#2	Explain the scientific background and rationale for the investigation being reported	4
Objectives	#3	State specific objectives, including any prespecified hypotheses	5
**Methods**			
Study design	#4	Present key elements of study design early in the paper	6
Setting	#5	Describe the setting, locations, and relevant dates, including periods of recruitment, exposure, follow-up, and data collection	6
None	#nut-5	Describe any characteristics of the study settings that might affect the dietary intake or nutritional status of the participants, if applicable.	N/A
Eligibility	#6a	Cohort study: Give the eligibility criteria and the sources and methods of selection of participants. Describe methods of follow-up. Case-control study: Give the eligibility criteria and the sources and methods of case ascertainment and control selection. Give the rationale for the choice of cases and controls. Cross-sectional study: Give the eligibility criteria, and the sources and methods of selection of participants.	6

None	#nut-6	Report any particular dietary, physiologic, or nutritional characteristics that were considered when selecting the target population.	N/A
None	#6b	Cohort study: For matched studies, give matching criteria and number of exposed and unexposed. Case-control study: For matched studies, give matching criteria and the number of controls per case.	N/A
Variables	#7	Clearly define all outcomes, exposures, predictors, potential confounders, and effect modifiers. Give diagnostic criteria, if applicable	7-9
None	#nut-7.1	Clearly define foods, food groups, nutrients, or other food components (e.g., preparation method, taxonomical descriptors, classification, chemical form).	8-9
None	#nut-7.2	When calculating dietary patterns, describe the methods to obtain them and their nutritional properties.	8-9
Data sources and measurement	#8	For each variable of interest give sources of data and details of methods of assessment (measurement). Describe comparability of assessment methods if there is more than one group. Give information separately for for exposed and unexposed groups if applicable.	7-9
None	#nut-8.1	Describe the dietary assessment method(s) (e.g., portion size estimation, number of days and items recorded, how it was developed and administered, and how quality was ensured); report if and how supplement intake was assessed.	8
None	#nut-8.2	Describe and justify food-composition data used; explain the procedure to match food composition with consumption data; describe the use of conversion factors, if applicable	N/A
None	#nut-8.3	Describe the nutrient requirements, recommendations, or dietary guidelines and the evaluation approach used to compare intake with the dietary reference values, if applicable	N/A
None	#nut-8.4	When using nutritional biomarkers, additionally use the STROBE-ME; report the type of biomarkers used and usefulness as dietary exposure markers	N/A
None	#nut-8.5	Describe the assessment of nondietary data (e.g., nutritional status and influencing factors) and timing of the assessment of these variables in relation to dietary assessment	8-9

None	#nut-8.6	Report on the validity of the dietary or nutritional assessment methods and any internal or external validation used in the study, if applicable	8
Bias	#9	Describe any efforts to address potential sources of bias	11
None	#nut-9	Report how bias in dietary or nutritional assessment was addressed (e.g., misreporting, changes in habits as a result of being measured, data imputation from other sources).	17
Study size	#10	Explain how the study size was arrived at	11
Quantitative variables	#11	Explain how quantitative variables were handled in the analyses. If applicable, describe which groupings were chosen, and why	9-11
None	#nut-11	Explain categorization of dietary/nutritional data (e.g., use of N-tiles and handling of nonconsumers) and the choice of reference category, if applicable.	8-9
Statistical methods	#12a	Describe all statistical methods, including those used to control for confounding	9-11
Subgroups and interactions	#12b	Describe any methods used to examine subgroups and interactions	10
Missing data	#12c	Explain how missing data were addressed	10
Loss to follow up	#12d	Cohort study: if applicable, explain how loss to follow-up was addressed. Case-control study: if applicable, explain how matching of cases and controls was addressed. Cross-sectional study: if applicable, describe analytical methods taking account of sampling strategy.	6-7
Sensitivity analysis	#12e	Describe any sensitivity analyses	11
None	#nut-12.1	Describe any statistical method used to combine dietary or nutritional data, if applicable.	9-10
None	#nut-12.2	Describe and justify the method for energy adjustments, intake modeling, and use of weighting factors, if applicable	N/A
None	#nut-12.3	Report any adjustments for measurement error (i.e., from a validity or calibration study).	N/A

Results

Participants	#13a	Report numbers of individuals at each stage of study—eg numbers potentially eligible, examined for eligibility, confirmed eligible, included in the study, completing follow-up, and analysed. Give information separately for for exposed and unexposed groups if applicable.	11
Non-participation	#13b	Give reasons for non-participation at each stage	6-7
Participant journey	#13c	Consider the use of a flow diagram	Supplementary Figure 1
None	#nut-13	Report the number of individuals excluded on the basis of missing, incomplete, or implausible dietary and nutritional data.	Supplementary Figure 1 & Page 6-7
Descriptive data	#14a	Give characteristics of study participants (eg demographic, clinical, social) and information on exposures and potential confounders. Give information separately for exposed and unexposed groups if applicable.	11
Missing data	#14b	Indicate number of participants with missing data for each variable of interest	Table 1
Follow-up time	#14c	Cohort study: Summarise follow-up time (eg, average and total amount)	11
None	#nut-14	Give the distribution of participant characteristics across the exposure variables, if applicable; specify if food consumption for the total population or consumers only was used to obtain results	11 & Table 1
Outcome data	#15	Cohort study: report numbers of outcome events or summary measures over time. Case-control study: report numbers in each exposure category, or summary measures of exposure. Cross-sectional study: report numbers of outcome events or summary measures.	Table 2
Main results	#16a	Give unadjusted estimates and, if applicable, confounder-adjusted estimates and their precision (eg, 95% confidence interval). Make clear which confounders were adjusted for and why they were included	Table 2 & Page 11

Category boundaries	#16b	Report category boundaries when continuous variables were categorized	7 & 9
Relative and absolute risks	#16c	If relevant, consider translating estimates of relative risk into absolute risk for a meaningful time period	N/A
None	#nut-16	Specify if nutrient intakes are reported with or without the inclusion of dietary supplement intake, if applicable.	N/A
Other analyses	#17	Report other analyses done—eg analyses of subgroups and interactions, and sensitivity analyses	12-13
None	#nut-17	Report any sensitivity analysis (e.g., exclusion of misreporters or outliers) and data imputation, if applicable	12-13
**Discussion**			
Key results	#18	Summarise key results with reference to study objectives	13
Limitations	#19	Discuss limitations of the study, taking into account sources of potential bias or imprecision. Discuss both direction and magnitude of any potential bias.	16
None	#nut-19	Describe the main limitations of the data sources and assessment methods used and implications for the interpretation of the findings	16
Interpretation	#20	Give a cautious overall interpretation considering objectives, limitations, multiplicity of analyses, results from similar studies, and other relevant evidence.	13-15
None	#nut-20	Report the nutritional relevance of the findings, given the complexity of diet or nutrition as an exposure.	15-17
Generalisability	#21	Discuss the generalisability (external validity) of the study results	16-17
**Other Information**			
Funding	#22	Give the source of funding and the role of the funders for the present study and, if applicable, for the original study on which the present article is based	18

Ethics	#nut-22.1	Describe the procedure for consent and study approval from ethics committee(s).	6
Data statement	#nut-22.2	Provide data collection tools and data as online material or explain how they can be accessed	18
